# History and Evolution of the Tuning Fork

**DOI:** 10.7759/cureus.51465

**Published:** 2024-01-01

**Authors:** Keerthi Eraniyan, Latha Ganti

**Affiliations:** 1 Biomedical Sciences, University of Central Florida, Orlando, USA; 2 Emergency Medicine & Neurology, University of Central Florida College of Medicine, Orlando, USA; 3 Medical Science, The Warren Alpert Medical School of Brown University, Providence, USA

**Keywords:** medical instruments, history of neurology, medical education, neurologic examination, tuning fork

## Abstract

This paper is a summary of the evolution of the tuning fork, a crucial part of the cranial nerves and auditory examination. The tuning fork is a two-pronged fork that resonates at a specific pitch when struck against a surface and has been proven to be incredibly useful in diagnosing and detecting hearing disorders. The tuning fork, an unassuming device in modern medicine, traces its origins back to an era when scientific understanding and medical diagnostics were in their nascent stages. Since its inception, this unpretentious instrument has played a pivotal role in the hands of healthcare practitioners, aiding in the diagnosis and assessment of various medical conditions. This paper embarks on a captivating journey through time to explore the origin, evolution, and significant milestones in the development of the tuning fork. From the first suggestion of differentiating hearing disorders to present-day tuning forks, this paper maps the different stages that the tuning fork has gone through and how its use has changed over time. Along the way, we will discover how the tuning fork has harmonized with music, medicine, and various scientific pursuits, enriching our understanding of sound and resonance while leaving an indelible mark on the course of human history. Delving into the historical context of its creation, this review uncovers the ingenious minds that birthed this innovative device and the pivotal moments that brought it to the forefront of human endeavors.

## Introduction and background

From its initial use as a musical tool to its incorporation into neurology, audiology, and beyond, the tuning fork's journey in medicine is a testament to human ingenuity and our unyielding pursuit of knowledge. This review aims to provide a thorough understanding of the tuning fork's historical significance in medicine, elucidating its continued relevance as a diagnostic aid in the ever-evolving landscape of healthcare. The tuning fork’s distinctive ability to produce a pure, consistent tone quickly garnered the appreciation of musicians. However, its applications transcended the realm of music and promptly found their way to the dynamic world of medicine. As medicine and technology advanced, so did the tuning fork's applications. The vibration and sound emitted by the tuning fork are used to observe sound conduction through bone [1]. Used frequently in Weber and Rinne tests to mainly diagnose hearing disorders, the tuning fork gradually acquired its pivotal role in auditory examination. The Weber test differentiates sensorineural hearing loss from conductive hearing loss [2]. The Rinne test assesses for conductive hearing loss [3]. Sensorineural hearing loss is hearing loss caused by damage to the neural pathway to the auditory cortex while conductive hearing loss is hearing loss caused by damage to the outer or middle ear. Using the same principle, the tuning fork is also used to detect fractures, which conduct sound less well through bone [4-6].

## Review

**Figure 1:**
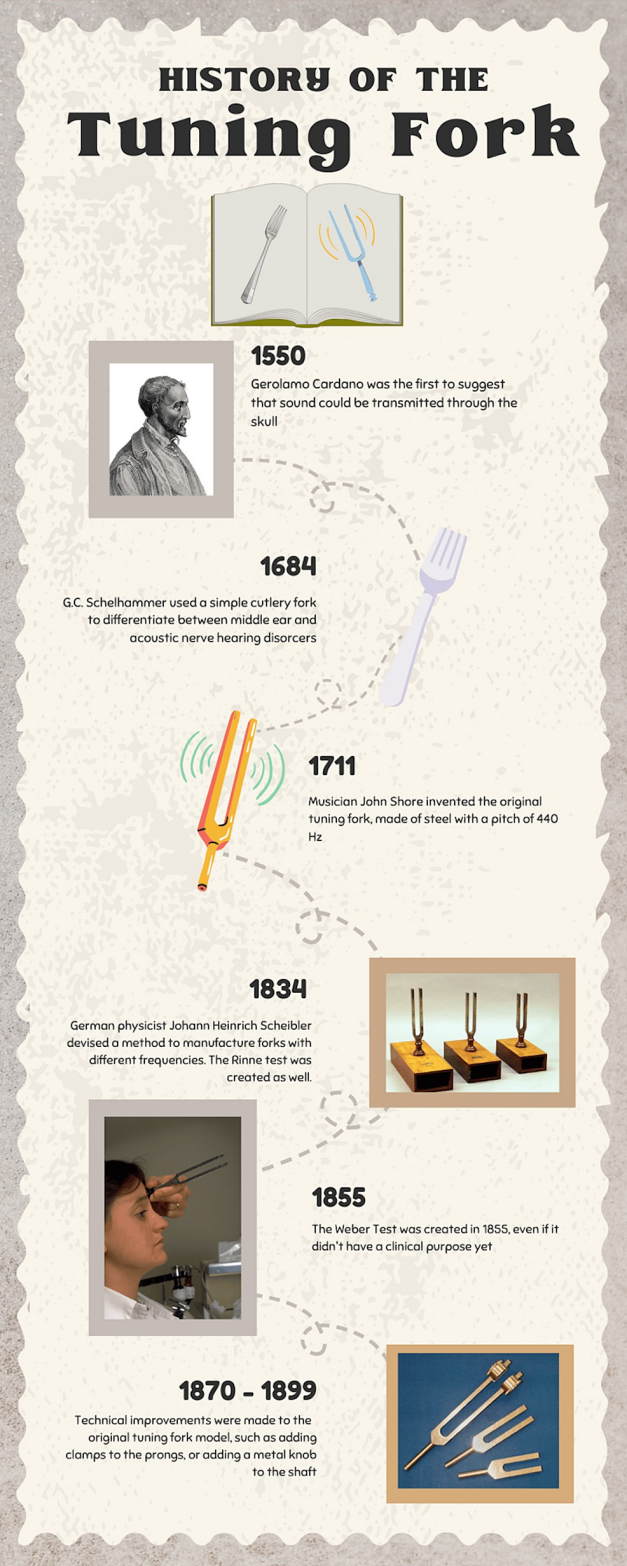
Infographic depicting the early history of the tuning fork Image created by Keerthi Eraniyan

The transformative historical narrative of the tuning fork unfolded almost 400 years ago. The tuning fork’s roots can be traced back to the sixteenth century when medical practitioners were beginning to realize the importance of observing bone conduction. It was 1546 when anatomist Giovanni Filippo Ingrassia first described the stapes, the auditory ossicle that conducts sound waves [7]. Four years later, Italian physician Geralamo Cardano proposed a novel concept that sound could be transmitted through the solid skull bone to reach the inner ear. This concept would later be known as bone conduction, though it had not been explicitly discovered yet (Figure 1) [8].

The first application of this phenomenon to differentiate hearing disorders was done by Hieronymus Capivacci in 1589. Before the invention of the tuning fork, medical researchers and practitioners had to innovate tools to mimic the vibration and consistent pitch that the tuning fork emitted. Capivacci placed one end of a rod against the vibrating string of a musical instrument called the zither and placed the other end between a patient's teeth. If the patient was able to hear the tone, he diagnosed them as having a hearing disorder resulting from damage to the tympanic membrane. If the patient was not able to hear the tone, the hearing disorder was diagnosed as damage to the innermost part of the ear. It was not until 1603 that bone conduction acquired its name, frequently referred to as “Ingrassia’s phenomenon.” Ingrassia had discovered that a vibrating table fork could be heard when pressed against the teeth, and this observation was only published in 1603, 23 years after his death. This is dubbed the birth of bone conduction. This idea of using a table fork to elicit pitch and vibrations was expanded on by G.C. Schellhammer, who ingeniously utilized a simple table fork to distinguish between different types of hearing disorders based on patients' responses to auditory vibrations. He held the stem of a vibrating tuning fork between a patient’s teeth. In one instance, the fork was touching the teeth, and in the other, it was simply held in the mouth. Through this test, Schellhammer discovered that sound conveyed through the teeth was transmitted through the cranial bones, not the Eustachian tube [8-11].

It was 1711 when the tuning fork was invented. Musician John Shore was originally a renowned trumpeter, who performed in the courts of King James II and played for George Frideric Handel. However, he split his lip after a concert and was never able to play the trumpet again. A musician at heart, Shore switched to playing the lute, a string instrument that required to be tuned regularly. However, to play this instrument, he encountered the challenge of tuning it accurately, an essential aspect for achieving harmonious melodies. With his ingenious spirit, Shore set out to create a solution, resulting in the invention of the tuning fork. This innovative device, with its ability to produce a consistent and precise pitch, allowed him to tune the lute with ease. Shore’s original tuning fork gave a pitch at 440 Hz, which is a musical note of A4. It began as a small steel fork with a stout stem and two flat, elongated prongs, which upon vibration produced a constant musical pitch. As Shore used his tuning fork before each of his concerts, the tuning fork found its place as a musical instrument employed in concert halls, churches, and chamber music ensembles throughout Europe. As the tuning fork became more widely used, the need for standardization of pitch became apparent. In 1834, German physicist Johann Heinrich Schreiber devised a method to precisely manufacture forks with different frequencies and created a set of 54 tuning forks ranging from 220 Hz to 440 Hz in 4 Hz intervals. The ‘scientific’ pitch was 512 Hz or a musical note of C5. This frequency came to be the standard pitch used in medicine [12].

The tuning fork reached its medical spotlight in 1834 when the Weber test was created. The Weber test was created to distinguish between conductive and sensorineural hearing loss in a patient with impaired hearing. That is, the patient had one ear that heard worse than the other ear. Weber placed a struck tuning fork at any point in the midline of the skull. If the tone was heard in the worse-hearing ear, the patient has conductive hearing loss. If the tone was heard in the opposite ear, the patient has sensorineural hearing loss. Despite its importance in diagnosing hearing disorders, the Weber test was not adopted by medical practitioners until almost a decade later, when French military physician Jean Pierre Bonnafont promulgated the Weber test as a diagnostic assessment [6-7].

While Weber’s test assessed hearing loss in both ears, Rinne’s test would come to assess hearing loss in one ear in 1855. Rinne’s test differentiated between air conduction and bone conduction. It was performed by holding a vibrating tuning fork to the mastoid bone behind the ear to conduct sound through the bone, and then in front of the external ear to conduct sound through the air. In a patient with healthy ears, Rinne realized that the tuning fork was heard better when merely placed next to the ear. In other words, one without a hearing disorder would have better air sound wave conduction than bone sound wave conduction. If bone conduction is better than air conduction, the patient has conductive hearing loss. If someone has impaired hearing but passes the Rinne test, they must have an auditory nerve disorder because the entire conduction apparatus is declared to be normal. It was with the creation of the Rinne and Weber tests that medical practitioners began to realize the unparalleled importance of the tuning fork [12-13].

In the late nineteenth century, the tuning fork was widely used in medicine. Certain technical improvements were made to the tuning fork to improve its utility. In 1870, Politzer installed clamps on the prongs of the fork to deaden overtones when it was struck. Könlg discovered in 1878 that the clamps could be shifted to vary the tone of the fork up to an octave. To ensure sufficient coupling with the skull when testing bone conduction, Lucae fixed a metal knob to the shaft in 1886. In 1899, a small hammer was fixed to the shaft to activate the fork when driven with a spring, and a wedge-shaped figure was drawn on the clamp to control the amplitude of the vibration. All of these improvements collectively shaped the tuning fork to what we now know as the current tuning fork [8].

While the tuning fork itself evolved minimally for the entirety of the twentieth century, it demonstrated its versatility when yet another use for it was found. During the early twentieth century early years, tuning forks found new applications in medicine when they were used to stimulate "vibration sense" on bony prominences. Vibration sense is the basic feeling of vibration against bone. This method, recognized as a crude test for neural pathways used in proprioception, which enables us to perceive body part location, movement, and action, was a concept developed by Landry, Bell, Bastian, Ferrier, and others during the nineteenth century. It played a vital role in diagnosing sensory and posterior column nerve disorders [7].

## Conclusions

The tuning fork’s history has proven to be a captivating journey through time, revealing its profound impact on various aspects of human knowledge and endeavor. From its humble origins as a musical instrument, the tuning fork's evolution into a versatile diagnostic tool in medicine stands as a testament to human ingenuity and adaptability. Over 400 years, the tuning fork has evolved from a simple metal rod pressed against a vibrating string to a revolutionary medical tool. Through the centuries, the tuning fork’s applications have been shaped and honed down, from understanding sound transmission through the skull to using it to differentiate hearing disorders. From the past to the present, the tuning fork's unwavering resonance serves as a reminder of the profound impact that a simple yet ingenious invention can have on the course of human history and the enhancement of human well-being.
